# Effect of Chitosan Nanoparticles as Edible Coating on the Storability and Quality of Apricot Fruits

**DOI:** 10.3390/polym14112227

**Published:** 2022-05-30

**Authors:** Eman H. A. Algarni, Ibrahim A. Elnaggar, Abd El-wahed N. Abd El-wahed, Ibrahim M. Taha, Huda A. AL-Jumayi, Sam M. Elhamamsy, Samy F. Mahmoud, Alaa Fahmy

**Affiliations:** 1 Department of Food Science and Nutrition, College of Science, Taif University, Al Hawiyah, P.O. Box 11099, Taif 21944, Saudi Arabia; eman1400@tu.edu.sa (E.H.A.A.); huda.a@tu.edu.sa (H.A.A.-J.); 2 Department of Horticulture, Faculty of Agriculture, Al-Azhar University, Nasr City, Cairo 11884, Egypt; abdelwahedabdelwahed485.el@azhar.edu.eg; 3 Department of Food Science and Technology, Faculty of Agriculture, Al-Azhar University, Nasr City, Cairo 11884, Egypt; ibrahimtaha164@azhar.edu.eg; 4 Department of Biochemistry, Faculty of Agriculture, Al-Azhar University, Nasr City, Cairo 11884, Egypt; sam79e@azhar.edu.eg; 5 Department of Biotechnology, College of Science, Taif University, P.O. Box 11099, Taif 21944, Saudi Arabia; s.farouk@tu.edu.sa; 6 Department of Chemistry, Faculty of Science, Al-Azhar University, Nasr City, Cairo 11884, Egypt

**Keywords:** apricots, coatings, chitosan nanoparticles, shelf-life, antimicrobial activity

## Abstract

Apricots are a fragile fruit that rots quickly after harvest. Therefore, they have a short shelf-life. The purpose of this work is to determine the effect of coatings containing chitosan (CH) as well as its nanoparticles (CHNPs) as thin films on the quality and shelf-life of apricots stored at room (25 ± 3 °C) and cold (5 ± 1 °C) temperatures. The physical, chemical, and sensorial changes that occurred during storage were assessed, and the shelf-life was estimated. Transmission electron microscopy was used to examine the size and shape of the nanoparticle. The nanoparticles had a spherical shape with an average diameter of 16.4 nm. During the storage of the apricots, those treated with CHNPs showed an obvious decrease in weight loss, decay percent, total soluble solids, and lipid peroxidation, whereas total acidity, ascorbic acid, and carotenoid content were higher than those in the fruits treated with CH and the untreated fruits (control). The findings of the sensory evaluation revealed a significant difference in the overall acceptability scores between the samples treated with CHNPs and the other samples. Finally, it was found that CHNP coatings improved the qualitative features of the apricots and extended their shelf-life for up to 9 days at room temperature storage and for 30 days in cold storage.

## 1. Introduction

Apricots, *Prunus armeniaca* L., a Rosaceae family member [1], is one of the most important stone fruits grown in Egypt. The ‘Canino’ apricot is the latest-maturing apricot fruit with a high monetary value in the Egyptian market. It also yields larger fruits than other varieties; however, the fruit is prone to chilling problems and has a short shelf-life [2]. Because it is prone to quick ripening and decaying after harvest, it is a fragile and climacteric fruit with a short shelf-life [3]. Apricots are typically harvested at a young age to ensure a longer shelf-life [4]. Chilling injury, wooliness, flesh translucency, flesh bleeding, and internal collapse may occur if apricot fruits are stored at low temperatures for an extended period [5,6]. Furthermore, the use of chemical agents on fruits is undesirable because consumers want safer and healthier meals that include less additives and synthetic substances [7].

Edible coatings have been extensively studied in recent years for the preservation of fruits and vegetables. Chitosan is one of the most promising biomaterials for the creation of edible coatings [8]. The most prevalent cationic polysaccharide is chitosan, which is a renewable resource and a low-cost biopolymer [9]. Furthermore, the United States Food and Drug Administration has accepted chitosan as a food additive [10,11]. The LD50 of chitosan in mice after oral administration is 16 g/kg body weight, which is practically equivalent to a family unit of sugar or salt. No symptoms have been reported in humans for up to 4.5 g/day of oral administration of chitosan [12]. Nanotechnology has been used in the improvement of coating technology for the preservation of fruits using diverse nano-systems, such as nanocomposites, nanoparticles, and nano-emulsions [13]. Chitosan nanoparticles (CHNPs) act as biopolymers as well as nanoparticles, including quantum size effects, and have a wide range of uses in antimicrobial treatments [14].

In the literature, nanoscale coatings have been reported to have other benefits, including reduction in moisture loss and hence retention of appearance, texture, and flavor. Such coatings act as a barrier for the gas exchange between fresh produce and its surroundings, reducing the rate of respiration and decay [15].

This impact was amplified when nanoscale coatings were used as a natural preservative on strawberries, apples, apricots, mandarins, pomegranates, and guavas after harvest [10,16,17,18,19,20]. This study evaluates the effectiveness of chitosan and chitosan nanoparticle formulations on the physical, chemical, and sensorial properties of apricot fruits and compares them to uncoated fruits (control) during storage to assess the improvement in the quality and extend the shelf-life of apricot fruits. It is the first time that the effect of chitosan as an edible coating on apricot fruit quality during storage periods and its efficiency as an antimicrobial agent under both cold storage and room temperature conditions are studied to assist in prolonging the life of apricot fruits in the market by reducing postharvest microbial spoilage in addition to reducing the need to apply fungicides, thus achieving a rewarding economic return.

## 2. Materials and Methods

### 2.1. Materials

Chitosan powder with a weight average of MW = 161,000 g/mol (degree of deacetylation: 77%) was purchased from Sigma-Aldrich. The other chemicals were bought from El-Nasr Pharmaceutical Chemicals Company, Cairo, Egypt.

Apricot (Prunus armeniaca) “Canino” fruits were picked from six-year-old trees grown in a private orchard located at El-postan, El-Behera Governorate, Egypt. The trees were planted at 5 × 5 m apart, budded on local apricot rootstock and grown in sandy soil under a drip irrigation system. Fruit samples were randomly collected at maturity in the middle of May from the four directions North, East, South, and West, and three levels (top, medium, and bottom) of the tree canopy. The fruits were uniform in size, color, and free from any visual defects. Fruits transported to the laboratory were without any signs of mechanical damage or deterioration.

#### Microorganisms’ Strains

Two Gram-negative bacteria (Escherichia coli and Salmonella typhimurium), two Gram-positive bacteria (*Staphylococcus aureus* and *Bacillus subtilis*), and two fungi strains (*Aspergillus niger* and *Aspergillus flavus*) were obtained from the Chemistry of Natural and Microbial Product Department, National Research Center, Giza, Egypt.

### 2.2. Methods

#### 2.2.1. Preparation of Chitosan Nanoparticles (CHNPs)

CHNPs were prepared by ionic gelation method. The CHNPs were prepared by the addition of a basic tripolyphosphate (TPP) solution to the chitosan (CH) acidic solution under stirring at room temperature [16]. Chitosan solutions were prepared by dispersing 1.0 g of chitosan in 100 mL aqueous acetic acid (1.0%) under stirring until the solution was transparent. Then, the pH was adjusted to 5.5 using NaOH (0.01 N). The sodium tripolyphosphate solution (1.0%) was then added to the chitosan solution dropwise during stirring. The formation of CHNPs started spontaneously via the TPP-initiated ionic gelation mechanism. The resulting suspension was kept at room temperature for 30 min with stirring. After that, Transmission electron microscopy (TEM) analyses were performed.

#### 2.2.2. Treatment Solution Preparation and Application (as Edible Coatings)

Fresh apricots were dipped for one minute in a sodium hypochlorite solution (250 mg L^−1^) then were air-dried (1 h). The fruits were separated into five categories: The first and second groups were immersed in CH (1.0 and 1.5%), respectively, while the third and fourth groups were immersed in CHNPs (1.0 and 1.5%, respectively), and the fifth group was immersed in distilled water as a control (uncoated). To prepare the coating solutions, glycerol were used as a plasticizer (1.5% *v*/*v*). After being immersed in the above solution for 3 min, the fruits, both treated and untreated, were then air-dried (2 h to ensure surface dryness).. All apricot samples were packaged in foam trays (L (100 mm) × W (100 mm) × H (40 mm)), then wrapped with poly-propylene stretch film with venting holes of 20 µm of thickness. Each group was then divided into two groups. The first was reserved at ambient temperature (25 ± 3 °C) and the second kept at cold temperature (5 ± 1 °C). The fruit samples were tested every five days during storage at 5 ± 1 °C, and every three days during storage at 25 ± 3 °C, until they were spoiled.

#### 2.2.3. Characterization of the Chitosan Nanoparticles

##### Transmission Electron Microscopic (TEM)

A High-Resolution Transmission Electron Microscopy (JEOL, JEM-1230, Tokyo, Japan) device with a 120 kV acceleration voltage was used to evaluate the size and morphology of the produced CHNPs.

##### Antimicrobial Activity

The antimicrobial activity of CHNPs against the microorganisms strain for *E. coli*, *S. typhimurium*, *S. aureus* and *B. subtilis* as well as *A. niger* and *A. flavus* was determined using the agar diffusion method, as described by [21,22]. In this method, sterile nutritional agar was prepared, and then the microbe strains were dispersed across the agar plate and left to dry. Under aseptic circumstances, two 5 mm diameter holes were made in each plate, and 100 mL of the nanoparticle suspension was then poured into the holes. The plates were incubated (for bacterial strains at 37 °C for 48 h and for fungal strains at 25 °C for 3–5 days) and then checked for evidence of a clean area surrounding the holes (inhibition zones). For each microorganism, the diameter of the inhibitory zones was measured and expressed in millimeters. Measurements were done in triplicate.

#### 2.2.4. Quality Criteria during Storage

Weight loss (%): The fruit was weighed at the beginning of the experiment up to 30 days. The findings were estimated as the percentage loss of weight according to the following equation:

Weight loss (%) = (Initial weight-weight at sampling date/initial weight) × 100.

Inspection of visual decay (%): At each time of analysis, visually decayed fruits from fruit samples were removed and the decay percentage was calculated as the number of decayed fruits. The number of decayed apricots, divided by the total number of apricots (100 pieces) and multiplied by 100, yielded the decay percentage (percent).

Total soluble solids (%): Apricots were homogenized and filtered. A hand refractometer (Atago Co., Tokyo, Japan) was used to measure total soluble solids (TSS) as a percentage. Measurements were done in triplicate.

Total acidity (%): Apricots fruit were cut into small pieces and homogenised in a grinder, and 5 g of ground apricots were suspended in 20 mL of distilled water and then filtered. The total acidity of the apricots were assessed using a pH-meter (Model-3505.UK) and titrated to pH 8.1 using 0.1 M NaOH. Total acidity was expressed as g of malic acid per 100 g of apricot weight. Measurements were done in triplicate. 

Ascorbic acid content: The concentration of ascorbic acid was measured using the official titrimetric method. Measurements were done in triplicate. 0.5 g of each apricot sample was macerated with 2 % (*v*/*v*) oxalic acid in a mortar and pestle, filtered and fill up to 50.0 mL with water. The mixture (10.0 mL) was titrated to pink with the 2,6-dichloro-dichopenol standard solution.

Carotenoids: Carotenoid pigments were extracted according to Akin et al. [23] with minor modifications. 5.0 g of sample was homogenized with 100 mL of methanol: petroleum ether/methanol (9:1 *v*/*v*), and the homogenized sample was then passed through a separating funnel. Sodium sulfite was used to filter the petroleum ether layer. Finally, pigments were spectrophotometrically quantified at 450 nm (Hitachi UV2800; Tokyo, Japan). The extinction coefficient was set at 2500, and the results were represented as β-carotene equivalents (milligrams of β-carotene per kilogram of fresh mass). Measurements were done in triplicate as mentioned above. The pigments concentration was calculated using 2500 as an average extinction coefficient for all carotenoids.

Lipid peroxidation: The concentration of malondialdehyde (MDA) in the fruit cellular membrane was estimated utilizing thiobarbituric acid reactive components [24] with minor modifications. About 4.0 g of fruit tissue was homogenized in 20.0 mL of 10% trichloroacetic acid and centrifuged for 15 min at 6000× *g*. The supernatant (2.0 mL) was combined with 6.0 mL of 0.6% thiobarbituric acid, then heated (in a water bath at 100 °C) for 20 min, quickly cooled (in ice bath), and centrifuged for 10 min at 6000× *g*. The amount of MDA was estimated using spectrophotometric measurements (at 450, 532, and 600 nm) of the supernatant absorbance. Where the measurements were done in triplicate.

Sensorial quality criteria: The sensory evaluation of the organoleptic properties of the apricot samples was performed by a panel of twenty-five trained members from the laboratory staff. Panelists were asked to rate the overall acceptability based on the criteria (general visual appeal, color, taste, and visible structural integrity appearance). The samples were evaluated during storage on a ten-point hedonic scale, with 9 indicating excellence and freshness, 7 indicating very good, 5 indicating good, 4 indicating acceptable, and 3 indicating fair. Measurements were assessed according to the method of Ezzat et al. [25]. Scores were then averaged, and a score ≥5 was considered acceptable for commercial purposes [26].

Shelf-life: According to Mondal [27], the shelf-life of apricots was measured by counting the days required for them to remain acceptable.

Analytical statistics: By examining the variance (one-way completely randomized design, ANOVA with three replications), the statistical analysis was carried out using the co-statistical software package (CoStat program, Version 6.311 (2005). CoHort Software, 798 Lighthouse Ave. PMB 320, Monterey, CA, 3940, USA). Duncan’s multiple range test was used to calculate the differences between means, with a significance level of *p* < 0.05.

## 3. Results

### 3.1. Characterization Results

#### 3.1.1. Chitosan Nanoparticle Morphology and Particle Size

**Figure 1:**
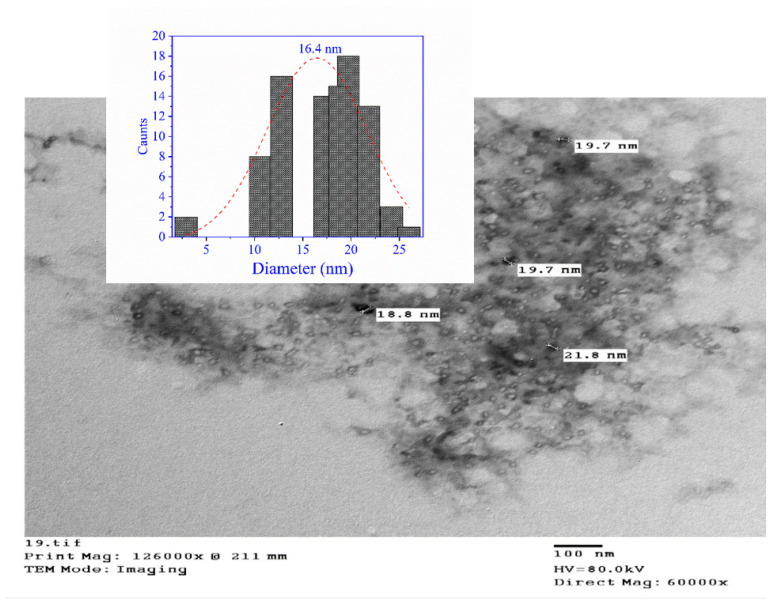
Micrograph of the chitosan nanoparticles.

The morphological features of the CHNPs were revealed by Transmission Electron Microscope (TEM) imaging, which showed that they have a roughly spherical form, a smooth surface with an average diameter of 16.4 nm (inset of Figure 1), as illustrated in Figure 1.

#### 3.1.2. Antimicrobial Activity of the Chitosan Nanoparticles

**Figure 2:**
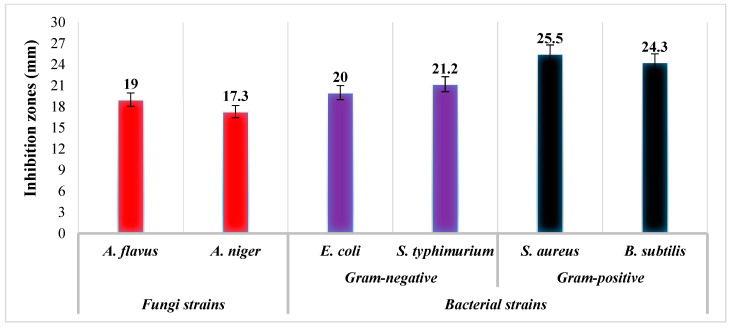
Effect of the chitosan nanoparticles on the inhibition of growth of the tested microorganisms.

The inhibitory zones generated on the medium were properly measured in mm and summarized in Figure 2. According to the findings, the inhibitory effect of CHNPs on the tested bacteria strains was larger than the inhibitory effect on the tested fungi strains. CHNPs had inhibitory zones ranging from 20.0 to 25.5 mm for bacterial strains, while for fungi strains were 17.3 to 19.0 mm. According to Duan [28], this strong bactericidal activity is due to a change in the cell permeability barrier caused by interactions between the positively charged chitosan and the negatively charged bacteria cell membranes.

Furthermore, *E. coli* and *S. typhimurium* were less susceptible to CHNPs (20.0 and 21.2 mm, respectively) compared to *S. aureus* and *B. subtilis* (25.5 and 24.3 mm, respectively). These findings match well with those reported by [29], who found that chitosan had a stronger effect on Gram-positive than Gram-negative bacteria. CHNPs, on the other hand, inhibit the growth of fungi strains, with inhibition zones of 19.0 mm for *A. flavus* and 17.3 mm for *A. niger*. The antimicrobial function of the films and coating layers inhibited microbial growth in foods and effectively increased shelf-life [30].

### 3.2. The Impact of Coatings on Apricot Fruit Quality during Preservation

#### 3.2.1. Weight Losses

**Scheme 1:**
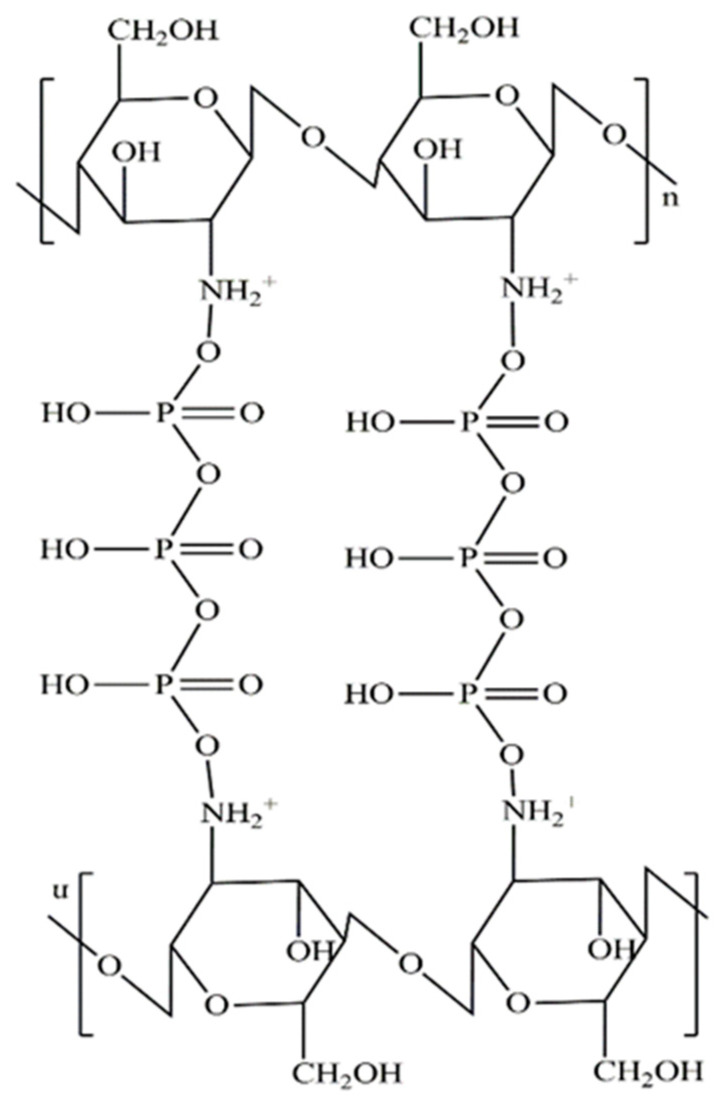
Suggested mechanisms of CHNP formation.

Weight loss is a good indicator of freshness and an essential factor of postharvest activities. Table 1 describes the effect of coatings on weight loss in apricots. During storage, all samples experienced a gradual increase (*p* < 0.05) in weight loss (%) with the coatings reducing weight loss (*p* < 0.05). At the end of storage at room temperature (9 days), the highest weight losses (*p* < 0.05) were seen in uncoated fruit (13.96%), whereas the lowest losses were reported in the sample coated with 1.5% CHNPs (6.75%). In terms of the effect of cold storage, the coated samples lost from 8.48 to 10.30% of their weight after 30 days. It is worth mentioning that the samples coated with 1.5% CHNPs had the least amount (*p* < 0.05) of loss (8.48%). Coating reduced water loss due to the formation of a barrier layer of biopolymers, which reduced water transfer from the foods [31]. In addition to the thickness of the CHNP film, which could be larger than that of the CH film due to the addition of sodium tripolyphosphate in CHNP, the nanoparticles in the coatings are responsible for creating a zigzag in the film structure (increasing the surface area) as well as a crosslinked-like structure (Scheme 1), which could hinder the passage of permeates, such as O_2_, CO_2_, and water vapor [32]. Moreover, the bonding and crosslinking in the structure of the chitosan nanocoating might be increased in the presence of tripolyphosphate molecules due to increased interaction between the oxygen of the polyanion and the hydrogen of the protonated amine in the chitosan through hydrogen bonding [33]. This slows down the rate of all vital processes and activities that take place inside the fruits.

#### 3.2.2. Percentage of Visual Decay

**Figure 3:**
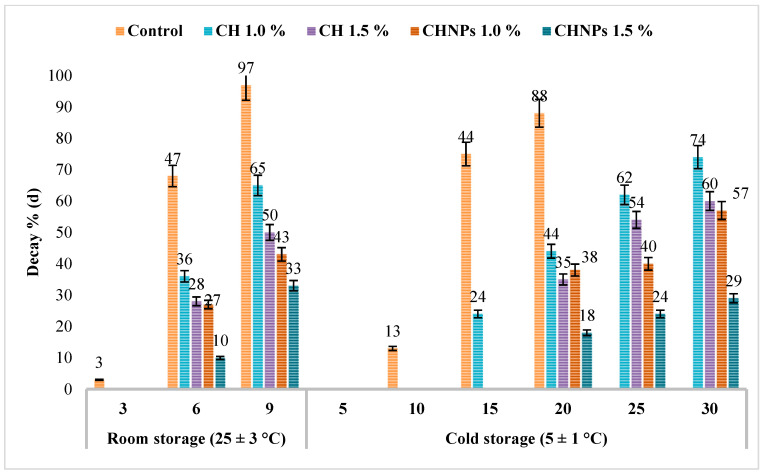
Effect of coating on apricot fruits’ visual decay percentage during storage.

One of the primary causes of postharvest losses in fruits and vegetables is decay. Figure 3 shows the influence of the coatings on the percentage of apricots that decayed. In general, the quantity of decayed apricots increased dramatically during storage. The coating treatments, on the other hand, lowered the percentage of apricot fruits that decayed. These findings match those of ref. [3], who found that the coatings were beneficial in preventing apricot fruit degradation at postharvest stages. The visual decay in the untreated apricot samples began with 3% on the third day of storage at room temperature, and grew with increasing storage times, reaching 47% on the sixth day and 97% at the end of the storage period (day 9). On the other hand, on the third day of room temperature storage, the coated apricots displayed no evidence of decay. On the sixth day of preservation, we saw visual decay in all coated apricots, where the coated samples with 1.5% CHNPs had the lowest decay percentage (10%), while the fruits coated with 1.0% CH had the greatest infection (36%). For samples coated with CH and CHNPs (at the levels of 1.0 and 1.5%), decay percentages were 65, 50, 43, and 33% at the conclusion of storage (day 9). The antimicrobial function in the coating layers inactivated microbial growth on the contact surface and effectively delayed the decay [34].

In cold storage, on the 10th day, the uncoated apricots displayed signs of decay (13%). During this time, there was no visible decay in the coated apricot samples. On the 15th day, the decay percent of the uncoated apricots increased to 44%. On the other hand, the coated apricots revealed no signs of decay, except for the sample coated with 1.0% CH, which exhibited 24% degradation. On the 20th day, all coated apricots started to show signs of decay, with 44, 35, 38, and 18% for the samples coated with (1.0 and 1.5 %) of CH and CHNPs (at the levels of 1.0 and 1.5%), respectively, compared to 88% for the untreated apricots on this day. The decay rates in the coated samples ranged from 24 to 62%. At the conclusion of cold storage (day 30), the decay percent in the coated samples ranged from 29 to 74%.

#### 3.2.3. Total Soluble Solids

Table 2 shows the total soluble solid (TSS) content of apricots as a function of coatings during storage. There were no significant variations in TSS among the coated samples throughout storage, but significant variations were observed between the uncoated and coated samples on the 3rd day at room temperature storage and from the 10th day in cold storage. In addition, as CH or CHNP concentrations increased, the increment rates in TSS content for the coated samples decreased.

Furthermore, the TSS content of all samples grew significantly with the lengthening of storage periods, except for the fruits treated with CH at 1.0% at room temperature storage and the coated apricots with CH 1.5% in cold storage, which exhibited a minor drop at the conclusion of the storage time. These findings are consistent with those of [35], who found that the TSS of fruits increased throughout storage due to the breakdown of starch into simple sugars or cell wall hydrolysis. Furthermore, when compared to the uncoated sample, the rate of TSS increment was lower in the coated samples, and the rate of fruit ripening was slower. This can be explained by a decrease in breathing. Furthermore, the coating slows the ripening of the fruit, preventing an increase in TSS concentration [36].

During storage at room temperature, the uncoated apricots had a higher TSS content increasing from 10.39 to 12.91% on the third day, compared to the coated samples, which had a lower increase rate (ranging from 1.16 to 1.51%) during the same previous storage period. TSS content for the apricot samples coated with CH (1.0 and 1.5%) and CHNPs (1.0 and 1.5%) at the end of storage was 14.82, 15.16, 15.35, and 15.22%, respectively, as shown in Table 2. On the other hand, in cold storage, the uncoated apricots had a TSS of 14.61% on day 15, while the coated apricots had TSS values ranging from 13.82 to 14.44% throughout the same storage period. It is worth noting that the TSS for the samples treated with 1.5% CH and CHNPs (1.0 and 1.5%) were 14.09, 14.75, and 14.72%, respectively, at the conclusion of storage (day 30).

#### 3.2.4. Total Acidity Content

Table 3 describes the effect of treatments on the acidity contents of the apricots. All treated and untreated apricots showed a gradual decrease (*p* < 0.05) of titratable acidity when stored for long periods. Additionally, while there were no significant differences in acidity contents amongst apricot samples at the start of storage, there were significant differences on day 9 of room temperature storage and day 10 and 15 of refrigerator storage. Upon first storage, the acidity content ranged from 1.04 to 1.06 g malic acid/100 g sample. These findings are consistent with a previous study that found apricot acidity of 0.7 to 3.0 g malic acid/100 g fresh sample [37,38]. At room temperature, with increasing storage periods, significant decreases in the total acidity content were detected in all the investigated apricot samples. This may be due to the consumption of organic acids in the breathing process with a slow decrease observed in the coated samples [39]. The apricot sample coated with 1.5% CHNPs had the smallest decline in total acidity (0.38%) at the conclusion of storage (day 9). The greater acidity drop in the uncoated strawberries could be attributed to the use of acids as precursors for metabolism throughout preservation. Nano-coatings could inhibit the respiration of samples and slow down the consumption of acid in the physiological metabolic activities of fruits, thus effectively slowing down the downward trend of titratable acid and extending the shelf-life of the fruits [40,41].

When it came to storing the apricot samples at a cold temperature, the uncoated apricots showed the greatest drop in TA content on day 10 (0.29%), while the apricots coated with 1.5% CHNPs showed the smallest reduction (0.6%) compared to the other samples, which varied from 0.10 to 0.16%. At the conclusion of cold storage (30 days), the fruits coated with CHNPs at 1.5% had the smallest decline in TA content (0.47%). This could mean that nano-membranes reduce sample respiration and slow acid intake in the physiological metabolic processes of the fruits.

#### 3.2.5. Content of Ascorbic Acid

Ascorbic acid is a relatively sensitive nutrient quality component that degrades quickly owing to oxidation during storage compared to other nutrients. Table 4 reveal that, as the storage period was extended, the content of ascorbic acid decreased progressively and significantly in all treatments. According to [42], the ascorbic acid concentration in apricots decreased continuously and considerably as storage period progressed. Additionally, according to ref. [43], these ascorbic acid declines after storage could be caused by the oxidation of dehydroascobic to diketogulonic acid. Reduced or delayed ascorbate oxidase activity could explain the action of chitosan and its nanoparticles. Additionally, as we can observe from the table, a significant variation in ascorbic acid content was identified among the tested treatments. During storage, the level of ascorbic acid in the apricots treated with CH or CHNPs was significantly higher than that in the control fruit. The CHNP treatments had the greatest concentration of ascorbic acid during storage.

Table 4 illustrates that the ascorbic acid concentration in fruit under cold storage was higher than that in the fruit stored at 25 ± 3 °C. The content of ascorbic acid of the tested apricot samples was 113.8 mg/kg for the fresh samples. These findings are consistent with those of reported in ref. [43]. They measured ascorbic acid values ranging from 18.0 to 132.0 mg/kg in fresh apricots. On the third day of room temperature storage, the ascorbic acid content of the uncoated fruit was 98.2 mg/kg, while the edible-coating apricots with CH (1.0 and 1.5%) and CHNPs (1.0 and 1.5%) contained 99.3, 101.1, 102.6, and 101.9 mg/kg, respectively. At the end of storage (9 days), the apricot samples coated with 1.0% CH had the highest loss (53.0 mg/kg) in ascorbic acid content, while the apricot samples coated with 1.5% CHNPs had the smallest reduction (45.1 mg/kg).

On the other hand, on day 15 of cold storage, the ascorbic acid content of the untreated apricots and those treated with CH (1.0 and 1.5%) and CHNPs (1.0 and 1.5%) was 61.1, 81.9, 83.8, 85.5, and 88.6 mg/kg, respectively. After 30 days of cold storage, the apricots coated with 1.5% CHNPs had the least amount of ascorbic acid loss (49.3 mg/kg). In general, coatings may slow down the ripening process and maintain high ascorbic acid levels by reducing oxygen transport. In comparison to the control fruits and the chitosan treatments during this investigation, CHNP treatments were much more successful in sustaining the ascorbic acid level of the fruits during storage.

#### 3.2.6. Carotenoid Content

Carotenoids are the pigments that give apricots their color, and their loss affects the overall quality and sensory acceptance of fresh fruit [44]. Table 5 shows the findings of the impact of the coatings on the carotenoid concentration in the apricot fruits. There were no significant variations in the carotenoid content among the uncoated and coated apricots during the early stages of storage, but there were considerable variances over the remainder of the storage period.

With longer periods of storage at room temperature, the carotenoid concentration increased. Apricots had a total carotenoid content of 111.6 mg/kg of β-carotene equivalent. These estimates are consistent with those obtained in other sources. The authors of [45] examined the total carotenoid content in apricots and discovered that carotenoid quantity varied amongst cultivars, ranging from 101.2 to 181.3 mg/kg of β-carotene equivalents.

On day 3, the untreated samples had the highest level of carotenoid content (119.6 mg/kg), whereas the apricots coated with 1.5% CHNPs had the lowest level (114.3 mg/kg). The levels of the other coated apricot samples ranged from 115.0 to 116.8 mg/kg. The uncoated apricots rotted on the sixth day of room temperature storage, while CH (1.0 and 1.5%) and CHNPs (1.0 and 1.5%) had carotenoid concentrations of 129.0, 131.7, 124.5, and 125.6 mg/kg, respectively. At the end of storage (day 9), a decline in carotenoid content was observed in the apricots coated with CH 1.0% to 115.1 and CH 1.5% to 124.3 mg/kg, meanwhile the carotenoid content in the apricots coated with CHNPs (1.0 and 1.5%) continuously increased to 128.5 and 144.7 mg/kg, respectively, with significant differences being observed.

On the other hand, after storage at cold temperature, the carotenoid content increased slightly to 128.5, 122.8, 121.3, 126.4, and 119.1 mg/kg on the 10th day, with significant differences between the untreated and samples treated with CH or CHNPs at the levels of 1.0 and 1.5%, respectively. On the 15th day, the untreated apricots had a drop in carotenoid content (118.8 mg/kg), but the coated apricots had an increase (*p* < 0.05) and reached 134.2, 129.1, 131.6, and 125.8 mg/kg, respectively, for the apricot samples coated with CH and CHNPs a t the levels of1.0 and 1.5%. The untreated sample spoiled on the 20th day, while the samples treated with 1.0% CH showed a decrease (*p* < 0.05) in carotenoid content (119.7 mg/kg) and the levels in the other coated samples increased. The storage period of the apricots treated with 1.0% CH ended on the 25th day, whereas the storage period of the other coated samples continued to increase past this time. At the end of the cold storage (day 30), the carotenoid content of the apricot samples coated with CH at 1.5% decreased to 137.5 mg/kg and that of the sample treated with 1.0% CHNPs to 152.3 mg/kg, whereas the carotenoid content of the apricots coated with CHNPs at 1.5% increased to 161.46 mg/kg. Moreover, carotenoid is highly sensitive to oxygen. The biopolymer coating reduced the oxygen diffusion to exposure with carotenoids in the fruits and delayed degradation [46].

#### 3.2.7. Lipid Peroxidation

**Figure 4:**
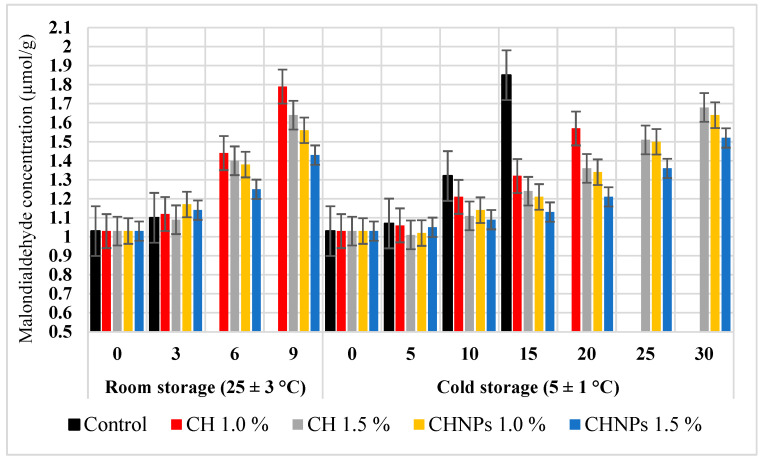
Malondialdehyde concentration (μmol g^−1^ FW) during storage.

Malondialdehyde (MDA) is commonly used as a measure of the structural integrity of plant membranes and is used to examine the progress of fruit ripening. It has also been employed as a direct sign of cell membrane damage and a measure of oxidative damage in cells during storage [47]. MDA determines the secondary products of oxidation process in the lipid components of foods, which directly affect the sensorial properties of foods during storage [48]. Figure 4 indicates the effect of the coatings and storage on the malondialdehyde levels in the apricot fruits. The cumulative MDA level in the coated and uncoated apricots increased with time as storage periods were extended. As a result of the ripening of the fruit, the MDA level increased during storage. At all storage times, the uncoated apricots had a considerably higher MDA concentration than the coated ones. The authors of ref. [5] reported that the MDA concentration of plums coated with chitosan was considerably lower than that of the untreated plums. Chitosan coating maintained membrane integrity by decreasing lipoxygenase activity and MDA buildup, as reported by ref. [49]. At the start of the sixth day of storage at room temperature, we noticed a considerable difference between the coated and uncoated apricots. At the end of the storage period (9th day), the samples coated with CHNPs had the lowest incremental rate of MDA (1.43 mol·g^−1^). In comparison to the storage at 25 ± 3 °C, increases in MDA concentration were slow when stored at 5 ± 1 °C. The incremental rate of MDA levels for the uncoated, CH-coated (1.0 and 1.5%), and CHNP-coated fruits (1.0 and 1.5%) were 0.82, 0.54, 0.65, 0.61, and 0.49 mol·g^−1^, respectively, at the end of storage period for each apricot sample.

#### 3.2.8. Sensory Quality Criteria

For consumers, the visual quality of apricots is the most important factor. The brightness or visual appearance of the fruit is positively connected with sensory acceptability and the inclination to purchase it [50]. The overall acceptability of the coated and uncoated samples was assessed and recorded in Table 6. Based on this table, most of the panelists assigned preference scores, such as “excellent and very good” on the first day, then “very good, good, or fair” on subsequent days of storage. The findings revealed that the coatings have a considerable impact on sensory evaluation criteria, with the nano-coating treatments outperforming the others. When compared to the uncoated fruit, the coated fruit with CH or CHNPs obtained higher marks. This could be because chitosan coatings change the color of the fruit giving it a glossy look, and the increased acceptance of the CH- and CHNP-coated fruits could be attributable to the flavor protection and spoiling prevention provided by the coatings [51].

At room temperature, the panelists first preferred coated fruit with CH and CHNPs as seen by the higher mean acceptance scores (from 8.46 to 8.59). All apricot samples were over the marketability limit (5.0 or more) until the third day. The uncoated apricots fell below the marketability and acceptability limits (4.0) on the sixth day of storage, recording 3.87. It is worth mentioning that, until the sixth day, all coated apricot samples were above the marketability limit. Table 6 shows that apricot fruits coated with CH at 1.0 and 1.5% were above the limit of acceptability and below the limit of marketability on the 9th day of storage, whereas the samples coated with CHNPs at 1.0 and 1.5% were above the limit of marketability. When apricots were stored at 5 ± 1 °C, the uncoated apricots decomposed on the 15th day of cold storage and did not reach the limit of marketability, but were over the limit of acceptability (4.25), which is consistent with the decay findings. All the coated apricot samples were above the limit of marketability up to the 25th day of cold storage, except for the sample coated with 1.0% CH, which dropped below the limit of marketability and limit of acceptability (3.38). Apricots coated with CHNPs remained within the marketable range on the 30th day of cold storage, but apricots coated with CH at 1.5% dropped below the marketability limit and over the acceptability level (4.03).

#### 3.2.9. Apricot Shelf-Life

**Figure 5:**
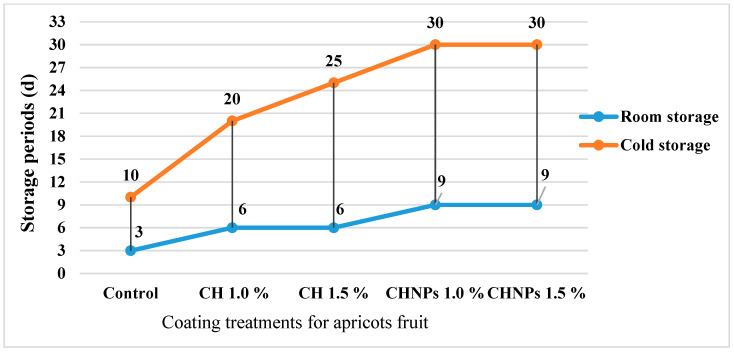
Shelf-life of the apricot fruits.

The ability to lengthen the shelf-life of the food chain is the most essential benefit of any antimicrobial treatment. The physical appearance, as measured by color retention, glossy appearance, and microbial decay, was used to determine shelf-life [52]. Figure 5 reveals that different apricot treatments can alter the spoiling profile and extend the shelf-life of the apricots. In general, the apricots stored at a cool temperature (5 ± 1 °C) had a longer shelf-life than those stored at ambient temperature (25 ± 3 °C). The uncoated apricots, for example, rotted after three days at room temperature and ten days at cold temperature. Thus, the samples treated by CH (1.0 and 1.5%) had a shelf-life of 6 days at room temperature, but these samples had a shelf-life of 20 and 25 days at cold temperature, as demonstrated in Figure 5.

For storage at room and low temperatures, the samples coated with CHNPs at 1.0 and 1.5% had a shelf-life of 9 and 30 days, respectively. The coatings modified the oxygen and carbon dioxide permeation, which affected the respiration of the fruits, and the antimicrobial activity delayed microbial growth, which reduced the rates of decay [53]. For apricot growers, these are important economic and encouraging implications.

## 4. Conclusions

The apricot is a climacteric fruit with a short postharvest storage life due to quality degradation. One of the most essential strategies to protect the quality of the apricot fruits is to coat them. Nanoscale materials have emerged as novel antimicrobial agents, where nanoparticles of chitosan were effective against tested pathogenic microorganisms. In this sector of the food industry, the use of CHNPs to enhance the shelf-life of apricots during storage appears to be quite promising. Apricots coated with CHNPs may be stored with a good quality for 30 days at 5 ± 1 °C and 9 days at 25 ± 3 °C, according to a complete comparison and evaluation. Chitosan and chitosan nanoparticle application after harvest control decay, maintains quality, and extends the shelf-life of the fruits. The overall acceptability scores were maintained in the fruits with nano-coatings compared to the uncoated samples, which lost their overall acceptability scores mainly due to the ripening speed and fungal infections, thus showing a poor quality.

## Figures and Tables

**Table 1:** Effect of coating with CH and CHNPs on the weight loss of apricot fruits during storage.

Treatment		Storage Period (Day) at 25 ± 3 °C			Storage Period (Day) at 5 ± 1 °C					
		3	6	9	5	10	15	20	25	30
**Control**		1.58 ^Ca^	8.00 ^Ba^	13.96 ^Aa^	1.30 ^Da^	3.97 ^Ca^	6.40 ^Ba^	8.86 ^Aa^	ND	ND
**CH** **%**	**1.0**	1.48 ^Ca^	5.55 ^Bb^	8.81 ^Ab^	1.22 ^Fab^	2.89 ^Eb^	4.55 ^Db^	5.90 ^Cb^	7.15 ^Ba^	10.30 ^Aa^
	**1.5**	1.42 ^Ca^	5.04 ^Bc^	7.73 ^Ac^	1.06 ^Fb^	2.78 ^Ebc^	4.33 ^Dc^	5.71 ^Cc^	6.87 ^Bb^	9.50 ^Ab^
**CHNPs** **%**	**1.0**	1.45 ^Ca^	4.91 ^Bc^	7.64 ^Ac^	1.14 ^Fab^	2.81 ^Ebc^	4.29 ^Dc^	5.47 ^Cd^	6.72 ^Bb^	9.06 ^Ab^
	**1.5**	1.50 ^Ca^	4.72 ^Bc^	6.75 ^Ad^	1.08 ^Fb^	2.62 ^Ec^	4.18 ^Dc^	5.04 ^Ce^	5.97 ^Bc^	8.48 ^Ac^

Mean values in the same row (as a capital letter) or column (as a small letter) with different letters are significantly different (*p* < 0.05). ND: not determined. For each parameter (row), different capital letters (A, B, C, D, E, F) in superscript indicate significant differences at *p* < 0.05 among storage treatments. For each column, different small letters (a, b, c, d, e) in superscript indicate significant differences at *p* < 0.05 among treatments for each measured parameter.

**Table 2:** Effect of coatings containing CH or CHNPs on the total soluble solids during storage.

Treatments		Storage Period (Day) at 25 ± 3 °C				Storage Period (Day) at 5 ± 1 °C						
		0	3	6	9	0	5	10	15	20	25	30
**Control**		10.39 ^Ba^	12.91 ^Aa^	ND	ND	10.39 ^Ca^	12.28 ^Ba^	14.41 ^Aa^	14.61 ^Aa^	ND	ND	ND
**CH** **%**	**1.0**	10.49 ^Ca^	12.00 ^Bb^	15.09 ^Aa^	14.82 ^Aa^	10.49 ^Ca^	12.19 ^Ba^	13.96 ^Aab^	14.44 ^Aab^	14.52 ^Aa^	ND	ND
	**1.5**	10.48 ^Ca^	11.79 ^Bb^	14.88 ^Aa^	15.16 ^Aa^	10.48 ^Ea^	11.95 ^Da^	13.68 ^Cb^	14.27 ^ABab^	14.39 ^ABa^	14.50 ^Aa^	14.09 ^BCb^
**CHNPs** **%**	**1.0**	10.50 ^Ca^	11.86 ^Bb^	14.90 ^Aa^	15.35 ^Aa^	10.50 ^Da^	11.99 ^Ca^	13.85 ^Bab^	14.34 ^ABab^	14.48 ^Aa^	14.59 ^Aa^	14.75 ^Aa^
	**1.5**	10.49 ^Da^	11.65 ^Cb^	14.75 ^Ba^	15.22 ^Aa^	10.49 ^Da^	11.82 ^Ca^	13.77 ^Bb^	13.82 ^Bb^	14.15 ^Ba^	14.53 ^ABa^	14.72 ^Aa^

Mean values in the same row (as a capital letter) or column (as a small letter) with different letters are significantly different (*p* < 0.05). ND: not determined. For each parameter (row), different capital letters (A, B, C, D, E) in superscript indicate significant differences at *p* < 0.05 among storage treatments. For each column, different small letters (a, b) in superscript indicate significant differences at *p* < 0.05 among treatments for each measured parameter.

**Table 3:** Effect of coatings on the total acidity of apricot fruits during storage.

Treatments		Storage Period (Day) at 25 ± 3 °C				Storage Period (Day) at 5 ± 1 °C						
		0	3	6	9	0	5	10	15	20	25	30
**Control**		1.04 ^Aa^	0.86 ^Ba^	ND	ND	1.04 ^Aa^	0.90 ^Aa^	0.75 ^Bc^	0.57 ^Cb^	ND	ND	ND
**CH** **%**	**1.0**	1.06 ^Aa^	0.95 ^Aa^	0.69 ^Ba^	0.46 ^Cb^	1.06 ^Aa^	0.94 ^ABa^	0.90 ^Bab^	0.79 ^BCa^	0.69 ^Ca^	ND	ND
	**1.5**	1.06 ^Aa^	0.94 ^Aa^	0.74 ^Ba^	0.58 ^Ca^	1.06 ^Aa^	0.97 ^ABa^	0.96 ^ABa^	0.83 ^BCa^	0.69 ^CDa^	0.60 ^DEa^	0.50 ^Ea^
**CHNPs** **%**	**1.0**	1.06 ^Aa^	0.94 ^Aa^	0.71 ^Ba^	0.61 ^Ba^	1.06 ^Aa^	0.99 ^ABa^	0.96 ^ABa^	0.85 ^BCa^	0.74 ^CDa^	0.63 ^DEa^	0.48 ^Ea^
	**1.5**	1.06 ^Aa^	0.98 ^Aa^	0.77 ^Ba^	0.68 ^Ba^	1.06 ^Aa^	1.03 ^Aa^	1.00 ^Aa^	0.92 ^ABa^	0.80 ^BCa^	0.70 ^CDa^	0.59 ^Da^

Mean values in the same row (as a capital letter) or column (as a small letter) with different letters are significantly different (*p* ≤ 0.05). ND: not determined. For each parameter (row), different capital letters (A, B, C, D, E) in superscript indicate significant differences at *p* < 0.05 among storage treatments. For each column, different small letters (a, b, c) in superscript indicate significant differences at *p* < 0.05 among treatments for each measured parameter.

**Table 4:** Effect of coating treatments on the ascorbic acid contents (mg/kg FW) of the apricot fruits.

Treatments		Storage Period (Day) at 25 ± 3 °C				Storage Period (Day) at 5 ± 1 °C						
		0	3	6	9	0	5	10	15	20	25	30
**Control**		113.8 ^Aa^	98.2 ^Bb^	ND	ND	113.8 ^Aa^	101.0 ^Ba^	82.3 ^Cc^	61.1 ^Dc^	ND	ND	ND
**CH** **%**	**1.0**	113.8 ^Aa^	99.3 ^Bab^	76.1 ^Cc^	60.8 ^Dc^	113.8 ^Aa^	102.3 ^Ba^	92.8 ^Cb^	81.9 ^Db^	70.7 ^Ec^	ND	ND
	**1.5**	113.8 ^Aa^	101.1 ^Ba^	78.8 ^Cb^	64.1 ^Db^	113.8 ^Aa^	102.9 ^Ba^	94.7 ^Ca^	83.8 ^Db^	74.8 ^Eb^	63.0 ^Fb^	51.2 ^Gb^
**CHNPs** **%**	**1.0**	113.8 ^Aa^	102.6 ^Ba^	77.4 ^Cbc^	65.4 ^Db^	113.8 ^Aa^	101.8 ^Ba^	94.4 ^Ca^	85.5 ^Db^	74.3 ^Eb^	64.9 ^Fb^	53.1 ^Gb^
	**1.5**	113.8 ^Aa^	101.9 ^Ba^	82.0 ^Ca^	68.7 ^Da^	113.8 ^Aa^	102.4 ^Ba^	96.0 ^Ca^	88.6 ^Da^	80.5 ^Ea^	75.1 ^Fa^	64.5 ^Ga^

Mean values in the same row (as a capital letter) or column (as a small letter) with different letters are significantly different (*p* ≤ 0.05). ND: not determined. For each parameter (row), different capital letters (A, B, C, D, E, F, G) in superscript indicate significant differences at *p* < 0.05 among storage treatments. For each column, different small letters (a, b, c) in superscript indicate significant differences at *p* < 0.05 among treatments for each measured parameter.

**Table 5:** Effect of the coatings on the carotenoid content (mg/kg FM) of the apricot fruits.

Treatments		Room Temperature Storage at 25 ± 3 °C				Cold Storage at 5 ± 1 °C						
		0	3	6	9	0	5	10	15	20	25	30
**Control**		111.6 ^Ba^	119.6 ^Aa^	ND	ND	111.6 ^Ca^	115.9 ^Ba^	128.5 ^Aa^	118.8 ^Bc^	ND	ND	ND
**CH** **(%)**	**1.0**	111.6 ^Ca^	116.8 ^Ba^	129.0 ^Aab^	115.1 ^Bc^	111.6 ^Ca^	115.0 ^Ca^	122.8 ^Bbc^	134.2 ^Aa^	119.7 ^Bb^	ND	ND
	**1.5**	111.6 ^Da^	115.0 ^Ca^	131.7 ^Aa^	124.3 ^Bb^	111.6 ^Ea^	116.6 ^DEa^	121.3 ^Dbc^	129.1 ^Cab^	138.3 ^Ba^	147.3 ^Aa^	137.5 ^Bc^
**CHNPs (%)**	**1.0**	111.6 ^Ba^	115.5 ^Ba^	124.5 ^Ab^	128.5 ^Ab^	111.6 ^Da^	115.8 ^Da^	126.4 ^Cab^	131.6 ^Cab^	142.6 ^Ba^	154.4 ^Aa^	152.3 ^Ab^
	**1.5**	111.6 ^Ca^	114.3 ^Ca^	125.6 ^Bab^	144.7 ^Aa^	111.6 ^Fa^	114.8 ^EFa^	119.1 ^Ec^	125.8 ^Dbc^	136.2 ^Ca^	148.7 ^Ba^	161.46 ^Aa^

Mean values in the same row (as a capital letter) or column (as a small letter) with different letters are significantly different (*p* ≤ 0.05). ND: Not determined, FW: Fresh weight.

**Table 6:** Effect of the coating treatments on the overall quality of the apricots during storage.

Treatments		Storage Periods (Day) at 25 ± 3 °C					Storage Periods (Day) at 5 ± 1 °C					
		0	3	6	9	0	5	10	15	20	25	30
**Control**		8.46 ^Ba^	8.95 ^Aa^	3.87 ^Cd^	ND	8.46 ^Ba^	8.93 ^Aa^	6.38 ^Cb^	4.25 ^Dd^	2.22 ^Ee^	ND	ND
**CH** **(%)**	**1.0**	8.59 ^Aa^	8.91 ^Aa^	6.63 ^Bc^	4.21 ^Cd^	8.59 ^Ba^	8.92 ^Aa^	8.89 ^ABa^	7.11 ^Cc^	5.62 ^Dd^	3.38 ^Ed^	ND
	**1.5**	8.58 ^Aa^	8.88 ^Aa^	6.94 ^Bc^	4.94 ^Cc^	8.58 ^Aa^	8.76 ^Aa^	8.84 ^Aa^	7.38 ^Bc^	6.27 ^Cc^	5.10 ^Dc^	4.03 ^Ec^
**CHNPs** **(%)**	**1.0**	8.58 ^Aa^	8.90 ^Aa^	7.27 ^Bb^	5.44 ^Cb^	8.58 ^Aa^	8.81 ^Aa^	8.91 ^Aa^	7.78 ^Bb^	6.80 ^Cb^	5.83 ^Db^	5.05 ^Eb^
	**1.5**	8.59 ^ABa^	8.84 ^Aa^	8.31 ^Ba^	6.26 ^Ca^	8.59 ^Aa^	8.71 ^Aa^	8.85 ^Aa^	8.26 ^Aa^	7.33 ^Ba^	6.49 ^Ca^	5.53 ^Da^

Mean values in the same row (as a capital letter) or column (as a small letter) with different letters are significantly different (*p* ≤ 0.05). ND: Not determined. For each parameter (row), different capital letters (A, B, C, D, E) in superscript indicate significant differences at *p* < 0.05 among storage treatments. For each column, different small letters (a, b, c, d, e) in superscript indicate significant differences at *p* < 0.05 among treatments for each measured parameter.
